# Versatility of Synthetic tRNAs in Genetic Code Expansion

**DOI:** 10.3390/genes9110537

**Published:** 2018-11-07

**Authors:** Kyle S. Hoffman, Ana Crnković, Dieter Söll

**Affiliations:** 1 Department of Molecular Biophysics and Biochemistry, Yale University, New Haven, CT 06520, USA; kyle.hoffman@yale.edu (K.S.H.); ana.crnkovic@yale.edu (A.C.); 2 Department of Chemistry, Yale University, New Haven, CT 06520, USA

**Keywords:** genetic code expansion, transfer RNA, synthetic biology, non-canonical amino acids, selenocysteine

## Abstract

Transfer RNA (tRNA) is a dynamic molecule used by all forms of life as a key component of the translation apparatus. Each tRNA is highly processed, structured, and modified, to accurately deliver amino acids to the ribosome for protein synthesis. The tRNA molecule is a critical component in synthetic biology methods for the synthesis of proteins designed to contain non-canonical amino acids (ncAAs). The multiple interactions and maturation requirements of a tRNA pose engineering challenges, but also offer tunable features. Major advances in the field of genetic code expansion have repeatedly demonstrated the central importance of suppressor tRNAs for efficient incorporation of ncAAs. Here we review the current status of two fundamentally different translation systems (TSs), selenocysteine (Sec)- and pyrrolysine (Pyl)-TSs. Idiosyncratic requirements of each of these TSs mandate how their tRNAs are adapted and dictate the techniques used to select or identify the best synthetic variants.

## 1. Introduction

**Figure 1:**
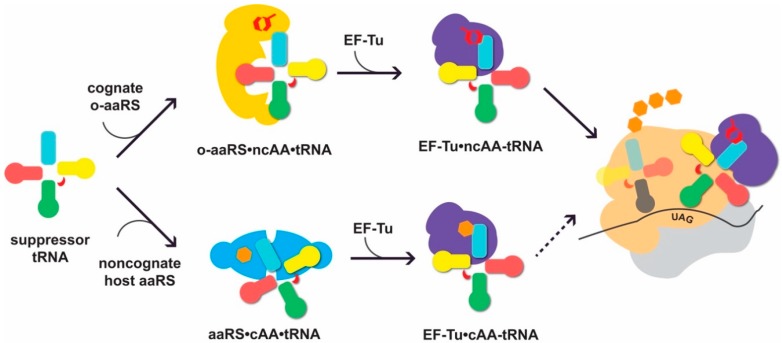
Suppressor transfer RNAs (tRNAs) interact with cognate orthogonal aminoacyl-tRNA synthetases (o-aaRSs) and the translational machinery of the host. For successful non-canonical amino acid (ncAA) incorporation, the suppressor tRNA needs to be recognized by its cognate o-aaRS and charged with the cognate ncAA (up). When not orthogonal, the tRNA can be erroneously recognized by an endogenous noncognate aaRS and aminoacylated with a canonical AA (cAA; down). The formation of cAA-tRNA can lead to cAA incorporation at the ribosome in response to UAG (depicted as a dotted arrow). Elements of the tRNA secondary structure are shown in light blue (acceptor stem), pink (D-arm), green (anticodon arm), red (variable loop), and yellow (T-arm). The o-aaRS is shown in yellow, noncognate, endogenous aaRS in cyan, elongation factor EF-Tu in purple, and the large and small ribosomal subunit in tan and light grey, respectively. NcAA is depicted as a red hexagonal shape, while the natural AAs are given in orange. The position of the UAG codon is indicated.

Genetic code expansion (GCE) involves the engineering of protein synthesis machinery to site-specifically incorporate non-canonical amino acids (ncAAs) into a desired protein [1,2]. This is routinely done by assigning the ncAA to recoded stop or sense codons and delivering the ncAA to the ribosome via a suppressor transfer RNA (tRNA). The successful charging of an ncAA to the suppressor tRNA and incorporation at a defined codon requires an aminoacyl-tRNA synthetase (aaRS)•tRNA pair to function orthogonally (restricting interactions with host tRNAs, aaRSs, or canonical amino acids; Figure 1). Non-canonical amino acids endow proteins with unique chemical and physical properties that make them useful for a wide range of applications. They serve as affinity tags, imaging probes, environmental sensors, post-translational modifications, are used for protein crosslinking, conjugation, and altering p*K*_a_ or redox potential [3].

The most versatile aaRS for incorporating ncAAs is pyrrolysyl-tRNA synthetase (PylRS). Naturally, PylRS attaches pyrrolysine (Pyl), the 22nd genetically encoded amino acid, to its cognate tRNA^Pyl^, a natural UAG suppressor. In archaea, PylRS is a single polypeptide chain; however, bacteria harbor a split protein where the C-terminal catalytic domain is only active in the presence of the N-terminal domain [4,5]. PylRS and its variants are polyspecific; to date they have facilitated the incorporation of over 100 ncAAs into proteins [6]. Moreover, PylRS•tRNA^Pyl^ pairs are used to engineer proteins with unique properties and functions in bacteria, viruses, insects, yeast, and animals [7,8,9,10,11].

Another valuable building block for protein engineering is the 21st amino acid, selenocysteine (Sec). Sec is a naturally occurring amino acid that resembles cysteine but has a selenol group instead of the thiol. Sec is found in the active site of redox enzymes of species that span all three domains of life, providing enhanced nucleophilic and reducing properties [12]. The site-specific incorporation of Sec can enhance enzyme activity when replacing cysteine (Cys), increase protein stability via diselenide bonds, and improve therapeutic peptides [13,14,15].

**Figure 2:**
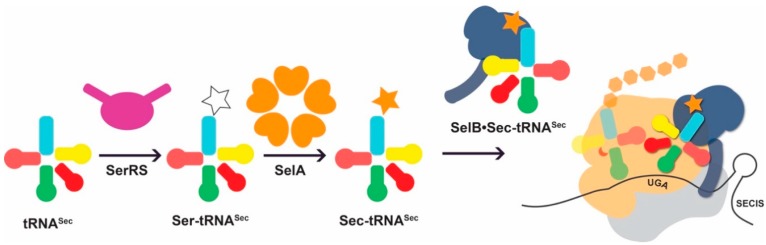
Idiosyncratic features of the natural Sec-incorporation pathway. tRNA^Sec^ is first misacylated with serine (white star) by seryl-tRNA synthetase (SerRS; purple). The intermediate, Ser-tRNA^Sec^ is a substrate for selenocysteine synthase (SelA) which converts the Ser moiety to Sec (orange star). Sec-tRNA^Sec^ is recognized by the Sec-specific elongation factor SelB (dark blue). In contrast to the general elongation factor EF-Tu, SelB approaches the ribosome bound to a Sec insertion sequence (SECIS), an RNA structure in its cognate mRNA. In this manner Sec-tRNA^Sec^ is directed to bind an upstream UGA codon and deliver Sec to the growing polypeptide chain.

While PylRS directly ligates an ncAA onto tRNA^Pyl^, there is no aaRS to form Sec-tRNA^Sec^. Rather, Sec is biosynthesized in a tRNA-dependent manner (reviewed in [4]). In bacteria, this first involves the charging of serine (Ser) by seryl-tRNA synthetase (SerRS) to form Ser-tRNA^Sec^, followed by the transfer of selenium from selenophosphate by selenocysteine synthase (SelA) for conversion to Sec-tRNA^Sec^ (Figure 2). In eukaryotes and archaea, Ser-tRNA^Sec^ is phosphorylated to form *O*-phosphoseryl-tRNA^Sec^ (Sep-tRNA^Sec^) by Sep-tRNA kinase (PSTK) [16], to which the phosphate group is displaced with selenophosphate by Sep-tRNA:Sec-tRNA synthase (SepSecS) [17,18,19]. Sec-tRNA^Sec^ delivery to the ribosome is aided by a selenocysteine-specific elongation factor (SelB in bacteria or EFSec in eukaryotes) [20,21]. Furthermore, the Sec insertion sequence (SECIS), an RNA structure in selenoprotein mRNA, recruits the SelB/EFSec-bound Sec-tRNA^Sec^ to the ribosome for the recoding of a UGA stop codon [22,23] (Figure 2). Given the diverse set of interactions and different mechanisms for Sec incorporation versus PylRS-mediated ncAA incorporation, the task of improving each system requires very different considerations.

When refining Sec- and Pyl-orthogonal translation system (OTS) components for GCE, it is ideal to produce a high amount of the ncAA-tRNA while retaining orthogonality and limiting the effects on cellular fitness. Heterologous aaRS•tRNA pairs for the OTS of a particular host organism are often imported from a different domain of life, since tRNA identity elements and substrate recognition are dissimilar enough to function orthogonally [24]. Moreover, the malleable active site of PylRS allows straightforward directed evolution methods to identify new ncAA-activating variants; however, these variants are polyspecific [25], and mutations that decrease the orthogonality must be selected against. Selenocysteine-OTSs are often used in bacteria or mammalian cells that already have the Sec pathway. Therefore, Sec pathway components are removed to prevent interaction with the OTS. Recent work in *Escherichia coli* has focused on improving the Sec incorporation efficiency and discovering EF-Tu compatible tRNA^Sec^ variants for selenoprotein expression without the requirement for SECIS in the coding sequence [26,27,28,29,30,31].

The production of Sec-tRNA^Sec^ is naturally inefficient compared to canonical aminoacyl-tRNA formation; SerRS serylates tRNA^Sec^ 100-fold less efficiently than tRNA^Ser^ [32]. It is likely that this kinetic inefficiency of SerRS correlates with the low demand for Sec incorporation; there are a limited number of proteins requiring Sec. Thus, the most challenging aspect of Sec-OTS engineering is to achieve efficient serylation by SerRS, as well as complete conversion to Sec-tRNA^Sec^ to ensure limited amounts of Ser misincorporation during selenoprotein expression [28]. Similarly, Pyl-tRNA^Pyl^ formation is inefficient compared to other aaRSs and PylRS has a moderate level of catalytic activity [25,33]. It has been a candidate for the evolution of enzyme variants with increased catalytic turnover, as well as more desirable ncAA specificity [34,35].

As a result of increasing ncAA-tRNA concentrations, the cellular levels of the PylRS•tRNA^Pyl^ pairs and components of the Sec pathway must be manipulated to out-compete host tRNAs or release factors for the targeted codon, while maintaining cellular fitness. Furthermore, altering the stoichiometry of the Sec-OTS components is important for the efficiency and homogeneity of selenoprotein production [26,29]. Thus, in addition to mutagenesis approaches to improving OTS interactions, the expression levels of each individual component are critical.

Due to their interactions with various parts of the translation machinery, tRNAs are central to achieving highly efficient ncAA incorporation, and both Sec- and Pyl-OTSs can be significantly improved through tRNA engineering. This is often accomplished through rational design, structure-guided mutagenesis, and random mutagenesis. Current molecular biology techniques facilitate the construction of large libraries of mutants, while combining positive and negative selection has been a successful approach to finding better variants. Here, we discuss the aspects of tRNA biology that should be carefully considered prior to OTS engineering and review the recent developments of Sec- and Pyl-OTSs with a main focus on tRNA design.

## 2. Aspects of Heterologous tRNA Expression

### 2.1. Identity Elements and Recognition

The identity elements of tRNAs are nucleotides and their modifications, which function as substrate recognition determinants. These determinants are found throughout the tRNA molecule and are essential for interaction with enzymes for aminoacyl-tRNA formation, as well as elongation factors. Moreover, tRNA recognition involves anti-determinant nucleotides and modifications to prevent the binding and charging of non-cognate tRNAs. In some cases, a single nucleotide mutation can change the tRNA identity and allow aminoacylation by a non-cognate aaRS [36]. Similarly, modifications may also confer identity; for example, m^1^G_37_ modification of tRNA^Asp^ in yeast is required to inhibit erroneous charging by ArgRS [37]. While some tRNAs (such as tRNA^Asp^ [38]), maintain their identity elements across all domains of life through divergent evolution, domain-specific idiosyncratic features required for aminoacylation are also present [39]. For this reason, aaRS•tRNA pairs can be transplanted from one domain of life to another and function orthogonally with respect to host aminoacylation.

Genetic code expansion designates a particular stop codon, or an “open” codon in genetically recoded organisms, for the insertion of an ncAA. Nonsense suppression is the most common way to insert ncAAs, since recoding is less detrimental to the proteome, given the low occurrence of stop codons. In this regard, the tRNAs of interest for GCE are typically those without identity elements in the anticodon, as their anticodons can be mutated to decode a stop codon of interest, while retaining aminoacylation capabilities. Conversely, if the active site of an aaRS is suitable for engineering ncAA substrate specificity, the anticodon binding domain can be evolved to recognize a nonsense suppressor tRNA [40,41,42,43].

The genetic code naturally expanded to include Sec and Pyl, through the recoding of UGA and UAG, respectively. However, Sec can be efficiently inserted at sense codons [44,45] and improving incorporation in a SECIS-independent manner is achieved through UAG suppression [26,28,29,31]. Anticodon mutations are sufficient to recode sense and stop codons with Sec and Pyl, since cognate SerRS and PylRS do not utilize identity elements in the anticodon loop of tRNA^Sec^ and tRNA^Pyl^. Thus, ncAA insertion can be easily directed towards a codon of interest using tRNA^Sec^ and tRNA^Pyl^, within the limitations of the host organism fitness and proteome perturbation.

### 2.2. Heterologous tRNA Modification and Maturation

Various factors influence the available pools of the aa-tRNA that can be used for peptide synthesis in the cell. These include amino acid and nutrient availability, tRNA expression and maturation (transcription, gene copy number, processing, and modifications), aaRS levels, and tRNA stability and degradation [46]. For GCE applications, the supply of ncAAs is controlled either by adding it in excess amounts to the growth medium or through metabolic engineering of the host organism (e.g., [47]). The biosynthesis and maturation of tRNA are more difficult processes to monitor and control. In *E. coli*, orthogonal tRNAs can be transcribed from “standard” constitutive and inducible promoters (e.g., *lpp*, *proK*, and P_BAD_). To mimic the coding sequences of bacterial tRNAs, the naturally absent terminal CCA sequence is added to the 3′-end of the archaeal tRNA gene. In contrast, to ensure proper processing in eukaryotes, the 3′-CCA sequence of bacterial orthogonal tRNA genes is typically removed.

While archaeal tRNAs in principle are not orthogonal to eukaryotic aaRSs (one exception being tRNA^Pyl^), bacterial tRNAs are utilized for GCE in eukaryotic hosts [1]. However, the normal transcription of tRNA genes in eukaryotic cells relies on RNA polymerase III, which recognizes A- and B-box promoter elements, present in the tRNA gene itself [48,49]. The majority of prokaryotic tRNAs lack such internal promoter sequences and the engineering of these o-tRNAs may lead to the artificial creation of A- and B-boxes in an o-tRNA variant (see below). To adapt the o-tRNAs of bacterial origin for transcription in yeast, two yeast Pol III promoters—the RPR1 promoter and the SNR52 promoter—have been shown to efficiently drive the expression of *E. coli* tRNAs [50]. Alternatively, a strong RNA polymerase II promoter with tandem tRNA repeats [51] or the yeast tRNA^Arg^ (used as a part of a dicistronic construct) fused upstream of the target tRNA [52] have also been developed.

Between 6.5% and 16.5% of tRNA nucleosides are post-transcriptionally modified, depending on the organisms [53], and over 100 different tRNA modifications have been identified (http://modomics.genesilico.pl/modifications/). Furthermore, tRNA processing is quite complex, sometimes involving intron splicing, trafficking to several subcellular locations [46], and even the ligation of two tRNA halves transcribed from different genes [54]. While the tRNAs used for GCE are orthogonal with respect to endogenous aaRSs, interactions with host modification and processing enzymes is required for function. The addition of tRNA modifications during biosynthesis is important for the stability [55], structure, and function of the molecule [56].

To ensure that an aberrant tRNA is not used for protein synthesis, tRNAs lacking certain modifications are targeted by nucleases for degradation. The nuclear surveillance turnover pathway ensures that a tRNA is properly modified during biosynthesis. For example, yeast pre-tRNA^iMet^ lacking m^1^A_58_ is polyadenylated by Trf4, which then triggers nuclease degradation by Rrp6 and the nuclear exosome [57,58]. The modifications m^7^G and m^5^C also play a role in tRNA stability. The rapid tRNA decay pathway (RTD) in yeast, involving 5′–3′ exonucleases Rat1 and Xrn1, targets mature tRNA that lack the m^7^G and m^5^C modifications [59,60]. These nucleotides provide an additional level of tRNA regulation and can be manipulated (through mutagenesis or the deletion of nonessential tRNA modifying enzymes) to prevent RTD-targeting and increase tRNA abundance, or for targeted degradation to decrease the toxicity of a suppressor tRNA [61].

The modification of tRNA nucleotides also affects codon–anticodon interactions, binding at the ribosomal A site [62], and ultimately the suppression efficiency that is desired for GCE applications. For instance, natural *E. coli* suppressors depend on the isopentenylation of adenosine 37 for full activity [63,64]. A genetic approach to addressing this issue involves monitoring ncAA incorporation and reporter protein yields across *E. coli* or yeast strain collections containing deletions and/or the overexpression cassettes of metabolic genes. Recently it was shown that the yield and specificity of *O-*phosphoserine incorporation is significantly improved by the deletion of cysteine desulfurase and the overexpression of *E. coli* dimethylallyltransferase (MiaA) and pseudouridine synthase (TruB) [65]. Furthermore, a yeast study involving the removal of modifications by single gene deletions from U34, U35, A37, U47 and C48 in the anticodon stem-loop impairs nonsense suppression, with the strongest effect observed for U34 and A37. Interestingly, the overexpression of eEF1a rescues the activity of an ochre suppressor tRNA (*SUP4*) and other non-suppressor tRNAs that lack modifications [66]. Thus, when designing suppressor tRNAs for GCE, tRNA modifications must be maintained or compensated for, such that tRNA stability and ncAA incorporation is not compromised.

## 3. When Amino Acid Biosynthesis is o-tRNA-Dependent: Challenges in tRNA^Sec^ Engineering

The biosynthesis of Sec-tRNA^Sec^ and its delivery to the ribosome is complex compared to the canonical amino acid pathway and involves several interactions with different portions of tRNA^Sec^. The major challenge in engineering tRNA^Sec^ for the more efficient incorporation of Sec is to improve serylation, while also having complete conversion of Ser-tRNA^Sec^ to Sec-tRNA^Sec^. In addition to this, the requirement of a SECIS sequence directly after UGA necessitates an EF-Tu-mediated Sec insertion pathway for the design and expression of selenoproteins in bacteria.

### 3.1. tRNA^Sec^ Interactions

**Figure 3:**
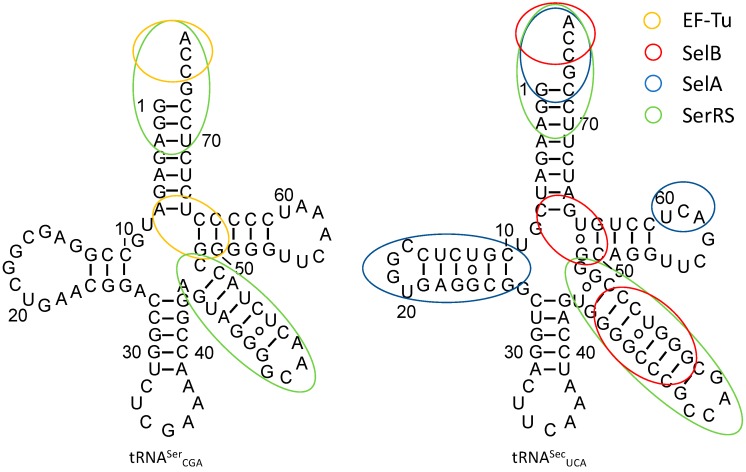
Secondary structure of *E. coli* tRNA^Ser^_CGA_ (**left**) and tRNA^Sec^_UCA_ (**right**). Identity elements required for accurate recognition by EF-Tu, SelB, SelA, and SerRS are given in orange, red, blue, and green, respectively.

The first step in Sec biosynthesis is the charging of tRNA^Sec^ with Ser by SerRS (Figure 2). SerRS lacks an anticodon binding domain, and changes to the anticodon stem-loop do not affect aminoacylation [67]. Rather, SerRS recognizes a long variable arm, a G73 discriminator base, and identity elements in the acceptor and D stems [68,69,70,71,72], which are conserved between tRNA^Ser^ and tRNA^Sec^ (Figure 3). These elements contribute to the structural features and shape of the tRNA and are important for the backbone and sequence-specific interactions for recognition by SerRS [68]. Of these features, the variable arm is most critical for aminoacylation. SerRS possess an N-terminal helical extension that interacts with the variable arm of tRNA^Ser^ and tRNA^Sec^, and properly orients the tRNA 3′ end for aminoacylation [68,73,74]. The overall length of the variable arm is more important than the sequence; the insertion of only one or two nucleotides in the variable arm of tRNA^Leu^ and tRNA^Tyr^, respectively, confers serylation activity and the deletion of a single base pair from the tRNA^Sec^ variable arm improves serylation 2–3 fold [32,70,75]. It is therefore not surprising that the variable arm accounts for the largest influence on the *K*_m_/*k*_cat_ of aminoacylation [67].

Identity elements of the tRNA^Sec^ extend beyond aminoacylation and include features of SelA and SelB interactions. Whereas canonical tRNAs have a 12-base-pair amino acid acceptor branch (7/5; consisting of a seven-base-pair acceptor stem and a five-base-pair T stem) that is recognized by EF-Tu/eEF1a, tRNA^Sec^ has a longer 13-base-pair acceptor branch (8/5 or 9/4). The deletion of a base pair from the acceptor stem of *E. coli* tRNA^Sec^ to resemble that of canonical tRNA^Ser^ abolishes UGA read-through with Sec [45], likely due to the disruption of the complex formation of tRNA^Sec^ with SelA and SelB [32]. In addition to the effects of the acceptor stem length on SelA recognition, nucleotides in the D arm form a unique structure compared to tRNA^Ser^, which is the basis of SelA-tRNA^Sec^ interaction [76].

Comparisons of SelB and EF-Tu complex structures show similarities of acceptor stem binding, but also unique domains and motifs that provide tRNA specificity. The N-terminal half of SelB consists of three domains, named D1, D2, and D3, that are analogous to those of EF-Tu [77]. D1 makes up the GTP-binding domain whereas, D2 and D3 consist of β-barrel-like and β-barrel structures for tRNA binding. Unique to SelB is a fourth domain (D4) comprised of four wing-helix motifs that recruit SelB to SECIS [78]. The structures of the SelB-Sec-tRNA^Sec^ complex obtained from single-particle cryo-electron microscopy depict how the linker region between D3 and D4 binds and distorts the variable arm of tRNA^Sec^, while an extended loop of D3 interacts with the acceptor and T stems [79]. In conjunction with the positively-charged SelB binding pocket, which provides affinity for the selenol group of Sec, and the altered variable arm orientation of tRNA^Sec^ compared to tRNA^Ser^, D3 and the linker between D3 and D4 of SelB provide Sec-tRNA^Sec^ specificity.

### 3.2. Converting tRNA^Sec^ Recognition from SelB to EF-Tu

The acceptor stem of the tRNA^Sec^ posed a challenge for engineering the EF-Tu-mediated Sec insertion. Although the binding specificity of tRNA^Sec^ can be switched from SelB to EF-Tu by shortening the acceptor stem [32], the eight-base-pair stem is important for the interaction with SelA. However, three base pairs in the T stem (49:65, 50:64, and 51:63) modulate the binding affinity of EF-Tu in a sequence-dependent manner [80]. In the same region, tRNA^Sec^ has different bases. Moreover, the last base pair of the acceptor stem and the first two base pairs of the T stem of tRNA^Sec^ are anti-determinants of EF-Tu complexed with GTP [81].

The first generation tRNA^Sec^ for EF-Tu recognition, named tRNA^UTu^ (U for Sec and Tu for EF-Tu), was designed using *E. coli* tRNA^Ser^ as a scaffold with the first seven base pairs of the *E. coli* tRNA^Sec^ acceptor stem [31]. The last base pair of the tRNA^UTu^ acceptor stem was transplanted from tRNA^Ser^ to eliminate the EF-Tu anti-determinant position. Serylation of tRNA^UTu^ was as efficient as canonical tRNA^Ser^, however, the Ser to Sec conversion was hampered, which led to ~30% Ser misincorporation. Nonetheless, tRNA^UTu^ was successfully used to site-specifically incorporate Sec into selenoproteins of bacterial and human origin in a SECIS-independent manner.

### 3.3. Improving Ser-to-Sec Conversion

Complementary approaches were taken to address the incomplete conversion of Ser-tRNA^UTu^ to Sec-tRNA^UTu^. *E. coli* tRNA^Sec^ was used as a scaffold for the random mutagenesis of the EF-Tu anti-determinant base pairs C7:G66, G49:U65, and C50:G64. A Sec-specific NMC-A β-lactamase reporter was selected as an efficient tRNA^Sec^ suppressor containing G7:C66, U49:G65, and C50:U64, which was named tRNA^SecUX^ [29]. In order to achieve nearly complete conversion of Ser to Sec, SelA expression was elevated, the tRNA^SecUX^ dosage was decreased, and PSTK was co-expressed to form a Sep-tRNA^SecUX^ intermediate, which would remain a substrate for SelA but not for EF-Tu prior to Sec conversion.

Other studies have built on tRNA^UTu^ to improve Ser to Sec conversion. Using the structure of *Aquifex aeolicus* SelA in a complex with *Thermus tengcongensis* tRNA^Sec^, twenty-nine different tRNA^UTu^ variants were rationally designed to include tRNA^Sec^ features that interact with SelA, while maintaining those that are required for EF-Tu binding. *E. coli* FDH_H_ was used as a Sec insertion reporter in a sensitive colorimetric assay to identify the best variant, named tRNA^UTuX^, which differed from tRNA^UTu^ at 11 positions [28]. Kinetic assays confirmed that the serylation of tRNA^UTuX^ was comparable to tRNA^UTu^ and tRNA^Sec^. Ser-to-Sec conversion was increased to 90%, reaching a similar conversion rate as *E. coli* tRNA^Sec^. Furthermore, Fourier transform ion cyclotron resonance (FT-ICR) mass spectrometry analysis confirmed Sec insertion by tRNA^UTuX^ into the selenoprotein, Grx1, but did not detect a peak corresponding to Ser insertion. More recently, tRNA^UTu^ was used as a template for the generation of chimeric molecules to improve Sec incorporation and selenoprotein yields. It was found that a single base change of A59C in tRNA^UTu^, generating a molecule named tRNA^UTu6^, resulted in the highest expression levels of human GPx1 and nearly 90% Sec incorporation [27].

### 3.4. Different tRNA^Sec^ Structures for the Optimization of Selenoprotein Production

In a bioinformatic search for novel tRNA^Sec^ molecules, a group of tRNAs with unusual cloverleaf structures were identified, named allo-tRNA [82,83]. Certain allo-tRNA species had tRNA^Ser^ identities and functioned as efficient amber suppressors with Ser [82]. Allo-tRNAs also contain SelA identity elements, but have a 12-base-pair acceptor branch as opposed to the 13-pair branch present in most tRNA^Sec^ molecules. SelA from *Aeromonas salmonicida* subsp. *pectinolytica* 34mel (*As*) was coupled with allo-tRNA for selenoprotein expression, since its cognate tRNA^Sec^ also possesses a 12-base-pair acceptor branch [26]. Allo-tRNA nucleotides in the D stem and acceptor stem were mutated to include *As* tRNA^Sec^ identities. In addition, the stoichiometry of allo-tRNA to *As* SelA was altered to ensure the complete sequestration of the tRNA for Ser-to-Sec conversion while also maintaining non-toxic levels of *As* SelA. Further optimizations and metabolic engineering efforts created a Sec-OTS consisting of allo-tRNA^UTu2D^, *As* SelD, *As* SelA, and *Treponema denticola* Trx1. Along with the high selenoprotein yields obtained with a Sec incorporation efficiency estimated at >90%, the stand-alone capabilities of this system make it ideal for use in other organisms [26].

## 4. Absolutely Orthogonal? Unique Features of tRNA^Pyl^

**Figure 4:**
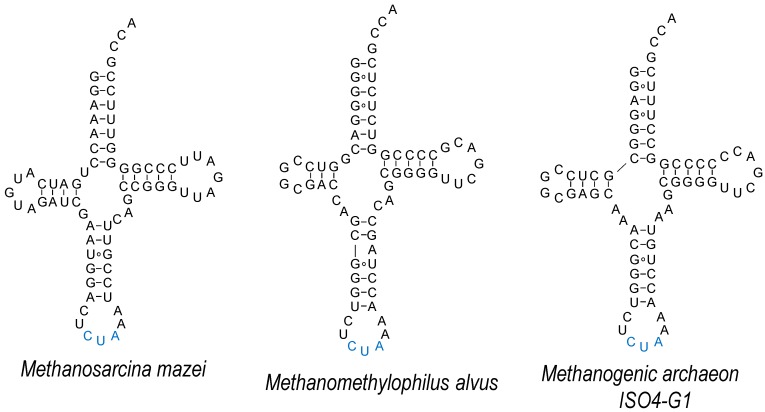
Structures of tRNA^Pyl^ belonging to mutually orthogonal Pyl-orthogonal translation systems (OTSs) [97,98].

Compared to the Sec system, the use of Pyl-OTS is comparatively less challenging, as its tRNA is orthogonal in the majority of model organisms used for GCE [84]; the enzyme is also orthogonal to both cellular tRNAs, as well as natural/canonical AAs [85]. Both bacterial and eukaryotic elongation factors accept tRNA^Pyl^, and the AA-binding pocket can be separately adapted to accept some bulkier ncAAs [86]. Attempts to advance ncAA delivery by tRNA^Pyl^ engineering include those aiming to improve its compatibility with the cellular machinery of the host. In *E. coli*, tRNA^Pyl^ was evolved by targeting the EF-Tu-binding regions [87], although the optimizing mutations present in tRNA^Pyl^_OPT_ may be more suitable for the delivery of one particular ncAA and less for the other (e.g., *N*^ε^-acetyllyine vs. 3-cyano-phenylalanine) [88]. The need to separately evolve an o-tRNA for a variety of “cognate” ncAAs or a variety of anticodons may require tunable binding by EF-Tu and the ribosome; while the stability of the EF-Tu•ncAA-tRNA complex reflects additive contributions by the ncAA and T-stem base pairs of the o-tRNA [80,89], the strength of codon–anticodon binding correlates with the nucleotide composition of the tRNA core [90]. For efficient expression in mammalian systems, a stabilizing mutation in the anticodon stem has been used (U29aC, Figure 4) [91,92]. By introducing elements conserved in human tRNAs, a better performing tRNA^Pyl^ was evolved. Mutations in the D-stem, D-loop, T-loop and the anticodon-stem U29aC proved to be indispensable for high activity [93]; compared to wild type tRNA^Pyl^, the use of this variant in HEK293 cells improved the incorporation of two ncAAs, *N*^ε^-carbobenzyloxy-lysine (Z-lysine) and *N*^ε^-(tert-butoxycarbonyl)-lysine (Boc-lysine). Interestingly, a chimera between mitochondrial (mt) tRNA^Ser^ and *Methanosarcina mazei* tRNA^Pyl^ improved the insertion of Boc-lysine selectively (C15) [93]. Earlier attempts at using mttRNA^Ser^ in *E. coli* failed, due to the lack of orthogonality [94]. The improved activity of M15 and C15 variants in mammalian cell lines may have to do with the appearance of the B-box in the T-arm of the variants; prokaryotic o-tRNAs are usually placed under the external promoter, such as U6, but the endogenous tRNAs are transcribed from internal A- and B-box promoters [48].

One of the distinct features of the Pyl system is the minimal variable loop of tRNA^Pyl^, which together with the T-loop forms a dipped surface [35,95] (Figure 4). From the crystal structure of the N-terminal domain of *M. mazei* PylRS it is evident that this minimalistic variable loop is a prerequisite for effective binding, as a larger variable loop would sterically clash with the N-domain [35]. In addition to *M. mazei*, *Desulfitobacterium hafniense* Pyl-OTS was employed in *E. coli*, either with its original N-terminal domain, or as a fusion with the recombinant (chimeric) N-domain of the archaeal system [96]. However, this system is not functional in mammalian cells [93]. As the N-terminal domain binds tRNA^Pyl^ with extremely high affinity [4], this element is likely to be an important contributor to (almost universal) Pyl-OTS orthogonality.

However, some organisms do not possess an equivalent to this N-domain [99], suggesting an alternative mode of recognition. This fact was recently exploited to develop mutually orthogonal Pyl-OTSs in *E. coli* [98] and mammalian cell lines [97,100]. Two PylRS enzymes that utilize the C-domain only (*Methanomethylophilus alvus* and methanogenic archaeon ISO4-G1) are highly active in *E. coli* [98]. Their cognate tRNAs retain some characteristic MmtRNA^Pyl^ features (such as the identity of the discriminator base G73, or the minimalistic D-loop) but also diverge in the nucleotide composition of the acceptor stem and in the probable structure of the anticodon stem (Figure 4). Given that the *M. mazei* and *M. alvus*/G1 systems are not fully orthogonal, rational engineering was employed in order to generate MatRNA^Pyl^ that would be recognized by MaPylRS and not MmPylRS. Variation of the nucleotide composition of the variable arm and/or its length allowed the generation of successful MatRNA^Pyl^ variants. Given the malleability of the PylRS active site, orthogonality to other OTSs [26,101], together with high activity of Pyl-OTSs in the bacteria and cells of higher eukaryotes [6], it is foreseeable that this dual encoding system will be commonly used.

The creation of multiple, mutually orthogonal OTSs is inherently related to the number of liberated codons that can be targeted for ncAA incorporation. In addition to UAG-directed incorporation, Pyl-OTS was also employed for ncAA incorporation in response to rare arginine (AGG) codons in *E. coli*, alone [102] or in tandem with *Methanocaldococcus jannaschii* Tyr-OTS [26]. A similar strategy was attempted in *Mycoplasma capricolum,* which possesses only six arginine CGG codons that should, in theory, facilitate the reassignment (Arg-to-Pyl) [103]. However, upon mutation of the tRNA^Pyl^ anticodon to CCG this almost universally orthogonal tRNA becomes a substrate for endogenous ArgRS. In conclusion, while the anticodon-blind recognition of PylRS allows the anticodon of tRNA^Pyl^ to be mutated into any nucleotide triplet, synonymous anticodons (such as CCU and CCG) can be recognized by host aaRSs with very different affinity, causing one tRNA^Pyl^ variant to lose its initial orthogonality.

## 5. Conclusions/Outlook

Improvements to OTSs have been emerging rapidly in recent years and are valuable for the accurate and efficient production of proteins containing ncAAs. The increasing amount of sequence data and bioinformatic/structural analyses reveal new molecules and novel mechanisms that help enhance each system. Moreover, advanced molecular cloning and directed evolution techniques help further shape the molecules that nature has provided into molecules that are better suited for the incorporation of ncAAs. tRNAs interact with each component of an OTS in the process of bringing the ncAA to the ribosome to insert a particular ncAA during peptide synthesis. For this reason, finding the best tRNA variant is critical for OTS developments. Our expanding knowledge of tRNA processing, maturation, and interaction mechanisms has guided tRNA engineering towards this goal. As we continue to learn more from nature and as technologies advance, it is conceivable that peptides with unique properties will be produced with significant industrial and medical implications.
